# PD-1/PD-L1 Inhibitor - Related Adverse Events and Their Management in Breast Cancer

**DOI:** 10.7150/jca.85433

**Published:** 2024-03-17

**Authors:** Chuqi Lei, Xiangyi Kong, Yuan Li, Huaiyu Yang, Ke Zhang, Zhongzhao Wang, Hu Chang, Lixue Xuan

**Affiliations:** 1 Department of Breast Surgical Oncology, National Cancer Center/National Clinical Research Center for Cancer/Cancer Hospital, Chinese Academy of Medical Sciences and Peking Union Medical College, Beijing, China.; 2 Administration Office, National Cancer Center/National Clinical Research Center for Cancer/Cancer Hospital, Chinese Academy of Medical Sciences and Peking Union Medical College, Beijing, 100021, China.

**Keywords:** breast cancer, immune checkpoints, adverse effect, PD-1/PD-L1, immunotherapy, management

## Abstract

As the positive results of multiple clinical trials were released, the Programmed cell death 1 (PD-1) and Programmed cell death ligand 1 (PD-L1) inhibitors emerge as the focus of integrative breast cancer treatment. PD-1/PD-L1 inhibitors are often used as a sequential agent to be combined with other agents such as chemotherapeutic agents, targeted agents, and radiation therapy. As multiple therapies are administered simultaneously or in sequence, they are prone to a variety of adverse effects on patients while achieving efficacy. It is a challenge for clinicians to maintaining the balance between immune-related adverse effects(irAEs) and treatment efficacy. Previous literatures have paid lots of attention on the adverse effects caused by immunosuppressive agents themselves, while there is a dearth of the research on the management of adverse immune effects during the combination of immunotherapy with other treatments. In this review, we discuss the overall incidence of irAEs caused by PD-1/PD-L1 inhibitors in combination with various types of treatments in breast cancer, including chemotherapy, CTLA-4 inhibitors, targeted therapy, and radiotherapy, and systematically summarizes the clinical management to each organ-related adverse immune reaction. It is important to emphasize that in the event of irAEs such as neurological, hematologic, and cardiac toxicity, there is no alternative treatment but to terminate immunotherapy. Thus, seeking more effective strategy of irAEs' management is imminent and clinicians are urged to raise the awareness of the management of adverse immune reactions.

## Introduction

Breast cancer has overtaken lung cancer as the most prevalent cancer in the world, with its incidence increasing at a rate of 3% per year, according to the “2020 Global Cancer Burden Update” published by the International Agency for Research on Cancer (IARC) 1, 2. Most patients with early-stage breast cancer can obtain good outcomes after standard traditional multidisciplinary treatment such as surgery, chemotherapy, endocrine therapy, targeted therapy, and radiotherapy. However, for some patients with advanced-stage or special pathological types such as Triple-negative breast cancer (TNBC) and c-erbB-2 positive breast cancer, the current treatment cannot prevent the progression of the disease effectively 3. Immunotherapy has made a great breakthrough in the treatment of advanced solid tumors by regulating the body's anti-tumor immune response and enhancing immune function to identify and eliminate tumor cells, which also provides new insights into the comprehensive treatment of advanced breast cancer 4,5,6,7,8,9,10.

Immunotherapy refers to the enhancement of anti-tumor immunity by blocking negative regulators of T-cell function present in immune cells and tumor cells through immune checkpoint inhibitors (ICIs). Although multiple regulators on T cells can serve as targets for ICIs, the most clinically widely studied are Programmed cell death 1 (PD-1) and its ligands. PD-1, a member of the CD28 family, is a transmembrane protein consisting of 268 amino acid residues that are widely present on the surface of immune cells such as T cells, B cells, macrophages, and dendritic cells 11. It possesses two ligands, PD-L1 (B7-H1) and PD-L2 (B7-DC). PD-L1 is the main ligand of PD-1 and is highly expressed on the surface of antigen-presenting cells (APCs) such as B cells and dendritic cells, as well as on the surface of many malignant tumor cells 12. Normally, the combination of PD-1 and PD-L1 can inhibit the proliferation and viability of CD4+ T cells and CD8+ T cells, preventing the immune response in vivo over-activation and suppressing autoimmune diseases. The same mechanism can be abused by tumor cells to reduce the immune cytotoxicity of T cells from the tumor microenvironment (TME) to evade immune surveillance 13. Consequently, ICIs can reinstate the tumor immune microenvironment by blocking the binding of PD-1 and its ligands between tumor cells and T cells and restoring the toxic effect of T cells to achieve tumor suppression.

To a certain extent, the immune microenvironment outside the tumor can be affected by the action of ICIs, which may trigger an auto-hyperimmune response and cause a range of toxic side effects 14. Such immune-related side effects induced by ICIs are called immune-related adverse effects (irAEs). Unlike conventional cytotoxic drugs and molecularly targeted drugs, the irAEs caused by ICIs can occur in any organ or tissue throughout the body, such as the skin, digestive organs, respiratory organs, thyroid gland, and pituitary gland. With the occurrence of 54%-76% and the mostly reversible nature of irAEs, the use of ICIs can be sustained under careful management 15, 16. However, moderate-to-severe irAEs can severely affect lung, liver, endocrine, and other organ functions, which may not only lead to treatment interruption and decreased efficacy but also reduce the patient's quality-of-life rating (QOL) and jeopardize life 15. To prolong the treatment duration and increase patient tolerance, early detection of irAEs and appropriate preventive or therapeutic measures are necessary.

So far, the Food and Drug Administration (FDA) of America has approved several ICIs targeting PD-1/PD-L1 for the treatment of various malignancies, among which promising results have been observed in the treatment of non-small cell lung cancer (NSCLC), melanoma, renal cell cancer (RCC) and hepatocellular carcinoma (HCC), with the response rate ranged from 50%-90% 17,18,19,20. In addition, there are currently 10 kinds of ICIs clinically used for malignant tumor treatment, including 7 anti-PD-1 antibodies and 3 anti-PD-L1 antibodies (**Table 1**). For breast cancer, several clinical trials have evaluated the efficacy of ICIs and shown positive results, such as IMpassion130 and KEYNOTE-012^21^, ^22^. Driven by these two clinical trials, PD-1/PD-L1 inhibitors became the first-line drugs for the treatment of advanced metastatic breast cancer. The subsequent gradual advancement of clinical trials KEYNOTE-086, KEYNOTE-119, KEYNOTE-355, KEYNOTE-173, KEYNOTE-522, I-SPY2 for pembrolizumab 23,24,25,26,27,28, IMpassion031, IMpassion131, IMpassion132 for atezolizumab 29,30,31, GeparNuevo 32 for durvalumab and other clinical trials have gradually clarified the application of ICIs in the integrated treatment of breast cancer. Because of the poor efficacy of single-agent applications, investigators have begun to focus on combination therapy with ICIs. Fortunately, the current clinical findings have already demonstrated the strength of combination therapy. While focusing on efficacy, the toxic side effects associated with immunosuppression should not be overlooked during the process of exploring ICIs in combination with other treatments for breast cancer. This review summarized the occurrence of irAEs in breast cancer patients treated with PD-1/PD-L1 inhibitors in combination therapies and concluded options and strategies for counteracting and attenuating immune-related toxicities, aiming at providing physicians with guidance and recommendations for immunotherapy safety management of breast cancer.

## 1. PD-1/PD-L1 inhibitor in combination with other therapy: status of treatment-related adverse effects

### 1.1 The irAEs of PD-1/PD-L1 inhibitors with chemotherapy in unresectable, locally advanced, or metastatic breast cancer

**Figure 1:**
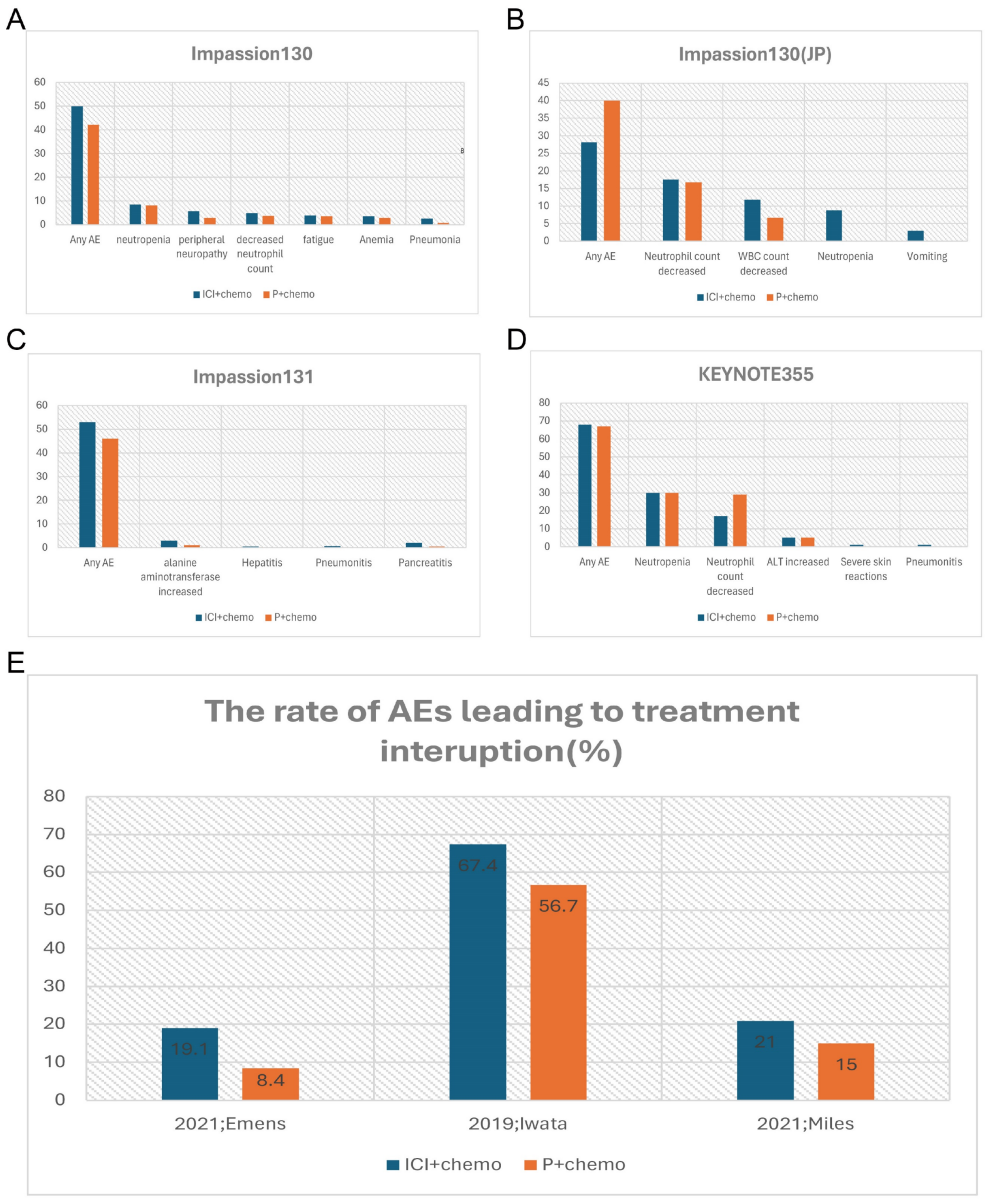
Comparison of incidence rate of irAEs (%) (grade3-4) and AEs (%) leading to treatment interruption between the combination group and the placebo group of the clinical trials. A, the incidence rate of irAEs in Impassion130 study; B, the incidence rate of irAEs in of Impassion130 Japanese subgroup study; C, the incidence rate of irAEs in Impassion131 study; D, the incidence rate of irAEs in KEYNOTE355 study; E, the rate of AEs leading to treatment interruption. irAEs = immune-related adverse effects; ICI = immune checkpoint inhibitor; P = placebo; AEs = adverse effects; JP = Japan.

Given that breast cancer relies on multidisciplinary treatment and that a few patients have been shown to benefit from monotherapy with ICIs in previous studies, more clinical studies have focused on the use of ICIs in combination with other treatment modalities (**Table 2**). Impassion130 was the first study to drive the combination of ICIs in breast cancer treatment. The major finding of this trial suggested a clinically meaningful overall survival and progress-free survival benefit with atezolizumab plus nab-paclitaxel in patients with PD-L1 immune cell-positive metastatic triple-negative breast cancer 33. In terms of safety, the most common Grade3-4 AEs are neutropenia (38 [8%] of 453 patients in the atezolizumab group vs 36 [8%] of 437 patients in the placebo group), peripheral neuropathy (25 [6%] vs 12 [3%]), decreased neutrophil count (22 [5%] vs 16 [4%]), and fatigue (17 [4%] vs 15 [3%]) (**Figure 1A**). Except for peripheral neuropathy (grade 3 only; 25 [6%] of 453 patients in the atezolizumab group vs 12 [3%] of 437 patients in the placebo group, the data between the atezolizumab group and placebo groups were not different, which may be explained by grade 3 peripheral neuropathy was taxane-related and cumulative. More treatment terminations have occurred in the combination group due to severe AEs than in the placebo group, with data of 30 [7%] vs 5 [1%] (**Figure 1E**). There were six cases (1%) of death in the combination group, one of which was ascribed to autoimmune hepatitis. With the longer follow-up time, no reduction in AE median time was observed during the combination period. Collectively, despite the absence of cumulative toxicity or new or late-onset safety signals, the use of ICIs increased the incidence of irAEs, which also resulted in compromising the treatment progress. The results of the IMpassion130 Japanese subgroup analysis suggested a lower incidence of treatment discontinuation caused by AEs, but a higher incidence of dose reduction and treatment interruption compared to the entire population of Impassion130 34 (**Figure 1B**). The finding supports the notion that appropriate treatment dose intervals could be a valid method to mitigate AE.

Instead of nab-paclitaxel, Impassion131 adopted paclitaxel as the chemo-agent with the result showing the addition of atezolizumab to paclitaxel neither improve investigator-assessed progression-free-survival (PFS) in the PD-L1-positive population nor the intent-to-treat (ITT) population at the primary PFS analysis with the same safety assessment 31 (**Figure 1C**). Whether atezolizumab impaired the physical ability to deliver paclitaxel remains uncertain and there is a lack of evidence of toxicity to explain the beneficial reduction.

Impassion132 29 is an ongoing randomized controlled clinical phase III trial, designed to evaluate the efficacy and safety of atezolizumab combined with first-line chemotherapy (capecitabine [mandated in platinum-pretreated patients] or gemcitabine/carboplatin) in early relapsing metastatic TNBC. Patients enrolled are those who have relapsed within 12 months of having completed the standard AT regimen of neoadjuvant chemotherapy, with a primary study focusing on overall survival (OS) in the ITT population. It is tempting to speculate that the beneficial effect of OS may be extrapolated to other chemotherapy regimens. Also, safety-related data is highly anticipated.

Another important phase III clinical trial named KEYNOTE355 24 has further confirmed the benefit of the anti-PD-L1 antibody combination chemotherapy in the treatment of locally advanced unresectable or metastatic TNBC patients. The validity is more pronounced in the PD-L1 positive (Comprehensive positive score, CPS ≥10) population. The incidence of irAEs was significantly higher in the combination group with an even higher rate of grade 3-4 irAEs, predominantly with severe skin reactions (**Figure 1D**). Despite the relatively manageable irAEs associated with anti-PD-L1 antibodies, Tolaney and colleagues noted that combining Pembrolizumab with Eribulin for ER-positive breast cancer not only failed to improve PFS, overall response rate (ORR), or OS, but also increased the incidence of serious adverse events 35. Specifically, there were two AE-related deaths in the combination group, which were attributed to progressive immune-related colitis, neutropenia, and sepsis. And the co-medication group had a higher incidence of fatigue, alopecia, and hepatitis. Due to the small sample size of this study, it remains unclear whether the lack of benefit in this trial is due to the disease subtype, pretreated population, the inclusion of patients with PD-L1-negative tumors, or the selection of the chemotherapy backbone, which requires further study.

### 1.2 The AEs of PD1/PDL1 inhibitors with neoadjuvant chemotherapy in early-stage breast cancer

**Figure 2:**
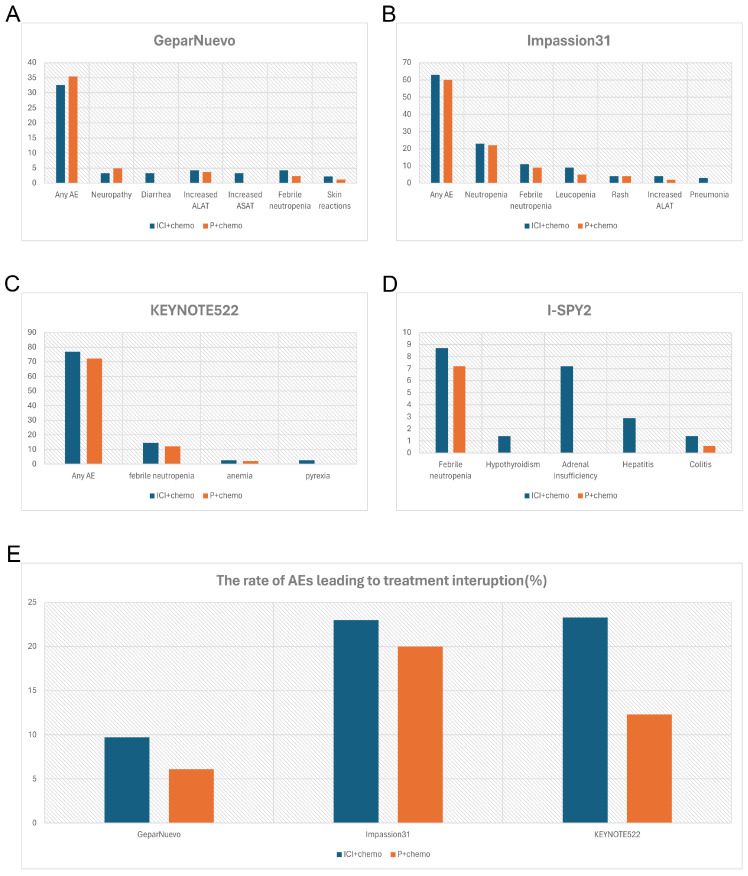
Comparison of incidence rate of irAEs (%) (grade3-4) and AEs (%) leading to treatment interruption between the combination therapy in NACT and placebo group. A, the incidence rate of irAEs in GeparNuevo study; B, the incidence rate of irAEs in of Impassion31 study; C, the incidence rate of irAEs in KEYNOTE522 study; D, the incidence rate of irAEs in I-SPY2 study; E, the rate of AEs leading to treatment interruption in the combination therapy in NACT. irAEs = immune-related adverse effects; ICI = immune checkpoint inhibitor; P = placebo; AEs = adverse effects; NACT = neoadjuvant chemotherapy.

Multiple studies have indicated superior treatment efficacy of ICIs in combination with early TNBC 36, 37 (**Table 3**). IMpassion031 is a representative study of neoadjuvant chemotherapy combined with ICIs in the treatment of breast cancer 30 (**Figure 2B**). The data of this trial provides compelling evidence that adding Atezolizumab to nab-paclitaxel followed by doxorubicin plus cyclophosphamide significantly improved pathological complete response rate regardless of PD-L1 status, with an acceptable safety profile. This finding contrasts with data from the IMpassion130 study in metastatic TNBC, in which atezolizumab showed benefit specifically in the PD-L1- positive population. One possible explanation for this phenomenon is that early TNBC populations with different PD-L1 statuses have a more powerful immune microenvironment to enhance anti-tumor responses 38. During this clinical trial, both the incidence of serious adverse events and adverse events of special interest were higher in the atezolizumab plus chemotherapy group, with Febrile neutropenia, pneumonia, and pyrexia being the most common (≥2% in either group), hepatic laboratory abnormalities, hypothyroidism, and infusion-related reactions being the most immune-related. Hypothyroidism was low-grade and clinically manageable. Similar results were seen in Foldi's trial 39 which targeted durvalumab plus weekly nab-paclitaxel.

In terms of efficacy, consistent with Impassion031, the results from KEYNOTE522 (**Table 3**) showed that the immune combination chemotherapy group increased the PCR of patients by 13.6% (64.8% vs 51.2%, p < 0.001), with a PCR of 68.9% vs 54.9% in the PD-L1-positive population [Combined Positive Score (CPS) ≥ 1%), and 45.3% vs 30.3% in the PD-L1-negative population, respectively 26. In KEYNOTE522, 32.5% of patients of the combination group developed severe AE, with febrile neutropenia (14.6% and 12.1%, respectively), anemia (2.6% and 2.1%, respectively), and pyrexia (2.6% and 0.3%, respectively) being the most common (**Figure 2C**). The addition of Pembrolizumab did not increase chemo-related toxicities such as myelosuppression, nausea, vomiting, renal insufficiency, and neuropathy, but significantly increased serious immune-related adverse events in the neoadjuvant phase.

GeparNuevo 32 has adopted a different regimen from the previous study. In this trial, the first dose of durvalumab was administered alone to patients 2 weeks before the start of nab-paclitaxel (window period). Results revealed a significantly higher PCR in the window period intervention group (61.0% vs 41.4%, p=0.035) (**Table 3**). The safety profile is in line with other PD-L1 inhibitor studies where thyroid dysfunction seems to be the leading toxicity 40,41,42 (**Figure 2A**). I-SPY2 pioneered the use of Pembrolizumab in the treatment of HR-positive/HER2-negative breast cancer 25. Finding that adding 4 cycles of Pembrolizumab to standard neoadjuvant chemotherapy could double PCR both in TNBC and HR-positive BC, which indicates that checkpoint blockade in women with early-stage, high-risk, HER2-negative breast cancer is highly likely to succeed in a phase 3 trial (**Table 3**). In this study, the addition of pembrolizumab mostly triggered endocrine irAEs, with a higher rate of thyroid abnormalities 41, 43 (**Figure 2D**).

Meanwhile, from the available data of GeparNuevo, Impassion031 and KEYNOTE522, adverse effects associated with the combination group resulted in a higher rate of treatment interruption (**Figure 2E**). This suggested that combining immunosuppressive agents during the neoadjuvant chemotherapy phase does not mitigate the adverse consequences of adverse reactions.

### 1.3 The AEs of PD-1/PD-L1 inhibitors with CTLA4 inhibitors

It has been recently proposed that multiple ICIs in combination may have a better performance in treating malignancy 44. Anti-cytotoxic T lymphocyte-associated protein 4 (CTLA4), which is one of the most common ICIs, has achieved remarkable pathological responses and relapse-free survival in 80% of patients with clinically detectable stage III melanoma in the neoadjuvant immunotherapy with anti-PD-1 monoclonal antibody 18. Several relevant clinical trials are being conducted on breast cancer (**Table 4**). A Phase I clinical trial led by M.D. Anderson Cancer Center (NCT03132467) aims at discovering the side effects of durvalumab and tremelimumab before surgery in treating patients with hormone receptor-positive, HER2-negative stage II-III breast cancer. Despite the efficacy, the trial was stopped early after 2 of 8 patients experienced grade 3 immune-related AEs with adrenal insufficiency (1/8), hyperthyroidism (1/8), and colitis (1/8). Another phase II clinical trial headed by Northwestern University (NCT02892734) focuses on the efficacy and the safety of nivolumab and ipilimumab when given as a combination in patients with metastatic recurrent epidermal HER2-negative inflammatory breast cancer. However, 2 of the 3 enrolled patients have presented with severe AEs: one with acute hypoxic respiratory failure and the other with pneumonitis. Despite the poor results of current clinical trials in the treatment of breast cancer, several ongoing clinical trial findings are worthy of anticipation: MOVIE (NCT03518606), a phase I/II multicenter, open-label study, focuses on evaluating a combination of metronomic oral vinorelbine plus anti-PD-L1/anti-CTLA4 Immunotherapy in patients with advanced breast cancer; ICON (NCT03409198), is a randomized phase IIb study evaluating immunogenic chemotherapy combined with Ipilimumab and Nivolumab in patients with metastatic hormone receptor-positive breast cancer. Data from these studies will guide the use of ICIs in combination with breast cancer.

### 1.4 The AEs of PD-1/PD-L1 inhibitors with Targeted therapy

HER2-positive breast cancer was a subtype of breast cancer with high aggressiveness and recurrent rate until the acquisition of anti-HER2-targeted drugs, which achieved a remarkable prognosis improvement. On top of that, a phase II clinical trial KATE2 explored the efficacy and safety of anti-HER2-targeted drugs in combination with ICIs in the treatment of advanced HER2-positive breast cancer 45. Despite the same prognosis, the addition of atezolizumab to trastuzumab emtansine resulted in a sizable increase in high-grade irAEs with a case of death due to hemophagocytic syndrome. Another clinical 1b/2 trial PANACEA 46 suggested that the addition of pembrolizumab to trastuzumab resulted in objective responses in patients with PD-L1-positive, but not PD-L1-negative tumors with manageable AE, further investigation of HER2-targeted agents plus atezolizumab in HER2-positive advanced breast cancer is warranted in PD-L1-positive patients.

In addition to HER2-positive breast cancer, Yuan and fellows facilitated a phase II clinical study 47 exploring pembrolizumab in combination with Enobosarm for treating androgen receptor-positive (AR+) metastatic TNBC. This study has given a promising prognosis of a clinical benefit rate (CBR) of 25%, which are higher than 9.5% of KEYNOTE86^23^. There were no grade 4 or above AEs observed and a minimum grade 3 AEs are: 1 (6%) musculoskeletal ache, 1 (6%) dry skin, and 1 (6%) diarrhea. Unfortunately, this trial was stopped early due to the withdrawal of drug supply for GTx-024, but data based on 16 evaluable patients suggested that the combination was well tolerated in heavily pretreated AR+ TNBC without PD-L1 pre-selected.

According to several preclinical models, poly adenosine diphosphate-ribose polymerase (PARP) inhibitors exhibited a synergistic anti-tumor ability with PD-L1 inhibitors regardless of the breast cancer susceptibility gene mutation rate or the PD-L1 expression 48. To further elucidate, an open-label, single-arm, phase 2 study named TOPACIO aimed at discovering whether the combination treatment of niraparib plus pembrolizumab would be a safe and effective therapy for patients with advanced or metastatic TNBC, was conducted 49, 50. The finding suggested a more pronounced benefit from combination therapy in patients with BRCA mutations, with an ORR of 47% and median PFS of 8.3 months, compared with an ORR of 11% and median PFS of 2.1 months in BRCA wild-type patients. The combination of niraparib and pembrolizumab did not result in new AE compared to monotherapy. Nausea, as the most common AE related to niraparib in breast cancer, remained at the same frequency. Hematologic was detected to be the most common grade 3 irAE and was consistent with the class effects of PARP inhibitors. These results revealed the safety profile of niraparib plus pembrolizumab in combination for patients with advanced or metastatic TNBC.

### 1.5 The AEs of PD-1/PD-L1 inhibitors with radiotherapy

Several studies have been carried out to show that the efficacy of PD-1/PD-L1 inhibitors is positively correlated with the expression of PD-L1 protein, and different induction modalities are capable to shift the PD-L1 expression on the tumor surface, while radiotherapy is one of the effective modalities to upregulation of PD-L1^51^, ^52^. Schweiger and collegues have comfirmed that radiotherapy modulated the immune suppression by increasing the PD-L1 expression of macrophages to affect Glioblastoma resistance 53. Another phase II clinical trial TONIC evaluated a sequential Nivolumab for metastatic TNBC after induction of changes in the tumor microenvironment by different cytotoxic agents or radiotherapy and also confirmed the ability to induce the TME of radiation 54. Another clinical study with a small sample revealed that the ORR of radiotherapy in combination with pembrolizumab in advanced TNBC was 17.6% (3/17), with 3 complete response (CR), 1 stable disease SD, and 13 progressive disease (PD)^55^. Although the present studies describing the role of radiotherapy in ICIs combination therapy were insufficient, it holds great promise for the future potential of this treatment option.

## 2. The overall incidence and clinical management of irAEs during the combination therapy

The pharmacodynamics of PD-1/PD-L1 inhibitors determined that their action on T cells is effective against tumors after activating the immune response of T cells to tumor cells, while an excessive autoimmune response may occur when T cells act on normal cells, resulting in irAEs^56^. Many of the irAEs are organ specific. A finding derived from a pan-cancer meta-analysis indicated that organ-specific irAEs include mainly hypothyroidism, pneumonia, colitis, hepatitis, and osteomyelitis, with pneumonia being the most common severe AE 40. In addition, general adverse events, such as fatigue, diarrhea, and rash, are known AEs associated with immune activation.

**Figure 3:**
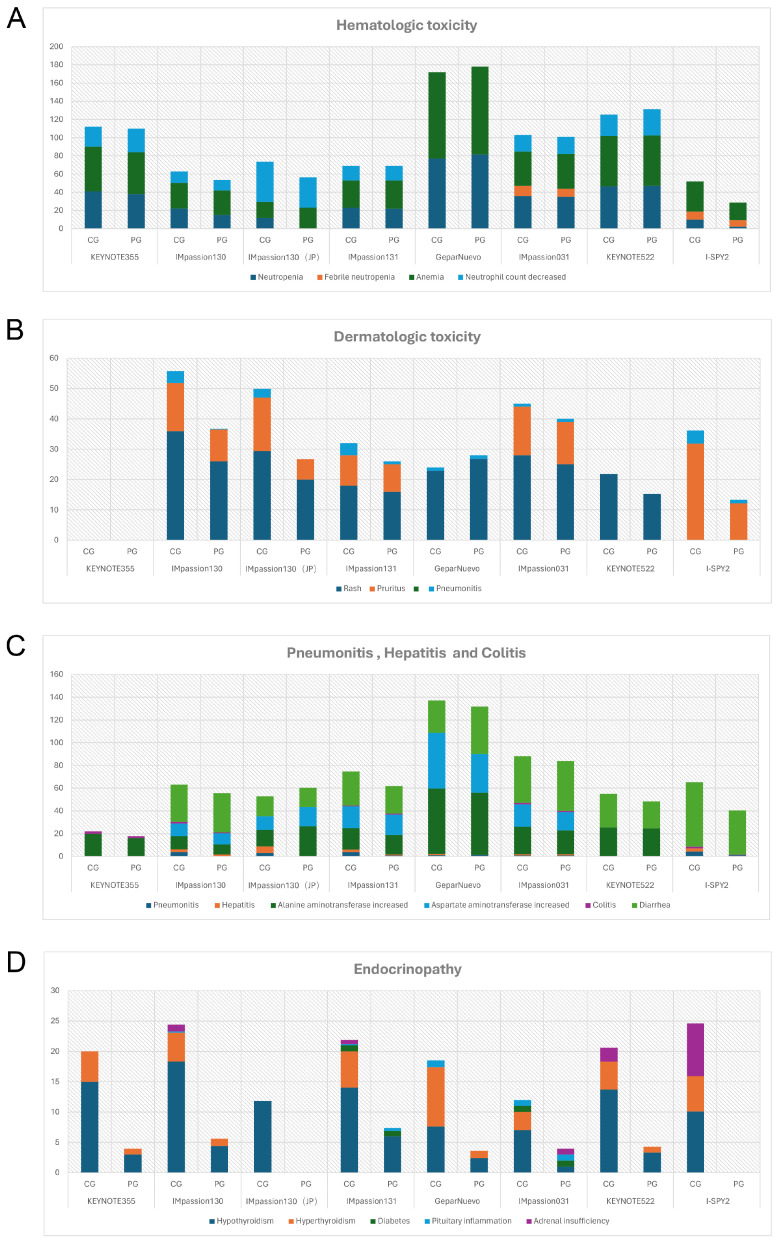
The Incidence Rate of Adverse Events (All grades) of Special Interest in Chemo-combined Clinical Trials (%). A, The rate of hemotologic toxicity. B, The rate of dermatologic toxicity. C, The rate of pneumonitis, hepatitis and colitis. D, The rate of endocrinopathy. CG = combination group; PG = placebo group; JP = the subgroup analysis of Japan.

The safety profiles of PD-1/PD-L1 inhibitors in combination therapy for breast cancer patients are generally manageable and tolerable. No new adverse effects have been reported as of the latest clinical trial results. The most common AEs of any grade in anti-PD-1/PD-L1 in combination therapy were fatigue (28%-87%), nausea (39%-79.7%), diarrhea (28.3%-56.5%), alopecia (33%-92.4%), and anemia (28.3%-94.6%). Different from monotherapy, pyrexia, and infusion-related reactions are relatively less likely to occur, accounting for approximately 20% of cases. Grade 3 or higher AEs were found in 78%-81%, 50%-63%, and 32.6% of patients receiving Pembrolizumab, Atezolizumab, and Durvalumab in combination therapy, respectively, were mostly immune-mediated, which might manifest as organ-specific autoimmune reaction 57. The focus is on hematologic abnormalities, severe skin reactions, organ-related inflammation (pneumonia, hepatitis, colitis), and endocrine disorders (**Figure 3**).

Since the irAEs occur over a wide period, patients who have received immunotherapy required close follow-ups. Once the suspicious symptoms of irAEs are detected, their severity should be promptly rated according to Common Terminology Criteria for Adverse Events (CTCAE) issued by the U.S. Department of Health and Human Services, and relevant clinical and laboratory tests should be performed to clarify the diagnosis 58. In general, patients with Grade 1 AE can continue treatment with ICIs under close monitoring, except for neurologic, hematologic, and cardiac toxicities that require prompt suspension of treatment 59. Patients with Grade 2-3 are advised to suspend ICIs until their symptoms or laboratory indicators drop to Grade 1 or lower, while most grade 4 irAEs require permanent termination of ICI therapy, except for some grade 4 endocrinopathies where hormone replacement therapy can be considered for ICI therapy again after the disease is fully controlled. The occurrence and severity of irAEs are unlikely to correlate with the drug dose of ICIs according to previous clinical trials, therefore a reduction in drug dosage is not recommended with Grade 2 and higher AEs, but rather a direct suspension of therapy. In addition to the suspension or discontinuation of immunotherapy, steroids are an effective treatment for irAEs: most Grade 2 and above irAEs can be controlled by oral or topical steroids. and the secondary side effects of steroids, such as increased opportunistic infections, are an important step in the management of irAE. The subsequent increase in some drug adverse effects such as opportunistic infections caused by steroids is also a very important step in irAEs management 60. For example, trimethoprim and sulfamethoxazole are given prophylactically to patients on long-term corticosteroids (>12 weeks) to prevent pneumocystis. The basal dose of oral corticosteroids is 0.4-1 mg/kg/day of prednisone or other similar drugs, which is appropriate for Grade 2 irAEs, while for Grade 3 or higher irAEs the dose needs to be increased to 5 mg/kg/day for 3-5 days. The following part is focusing on the management strategies of each organ-related immune adverse reaction (**Table 5**).

### 2.1 Hematologic toxicities

**Figure 4:**
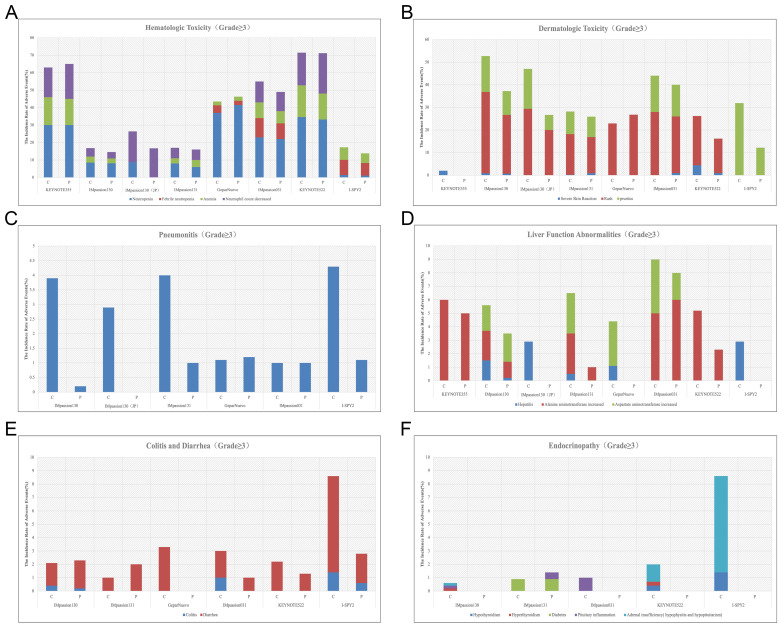
A, The incidence rate of hematologic irAEs (grade≥3) during the treatment of ICIs combination therapy. B, The incidence rate of dermatologic toxic effects (grade≥3) during the treatment of ICIs combination therapy. C, The incidence rate of Pneumonitis (grade≥3) during the treatment of ICIs combination therapy. D, The incidence rate of abnormal liver function (grade≥3) during the treatment of ICIs combination therapy. E, The incidence rate of diarrhea and colitis (grade≥3) during the treatment of ICIs combination therapy. F, The incidence rate of Endocrinopathy (grade≥3) during the treatment of ICIs combination therapy. (C = combination group; P = placebo group).

Hematologic irAEs occur occasionally during the treatment of ICIs combination therapy and the severity range from mild to severe. Previously reported diseases such as asymptomatic cytopenia, immune thrombolytics purpura, autoimmune hemolytic anemia, acquired hemophilia, and disseminated intravascular coagulopathy are comprehensive 61. In clinical trials of ICIs combination therapy for breast cancer, febrile neutropenia and anemia were the most common Hematologic irAEs. In IMpassion130, IMpassion 031, KEYNOTE355, and KEYNOTE 522, neutropenia and decreased neutrophil count were the most common grade 3-4 adverse effects (**Figure 4A**). Notably, anemia is the other most common grade 3-4AE in KEYNOTE 355 and KEYNOTE 522, while anemia is rare or even no patient shows symptoms of anemia in IMpassion 031 or IMpassion 130. Suggests that pembrolizumab is more likely to cause the development of anemia, while atezolizumab has no concerns in this regard.

In cases of mild hematologic abnormalities, patients can continue immunosuppressive therapy under close observation, but in cases of severe hematologic abnormalities, appropriate management is required. Generally, the condition will improve with the suspension of ICIs and corticosteroids for supportive management, and transfusion of blood products as needed. However, in isolated cases, cytopenia is ineffective to the cessation of therapy or steroid treatment, in that way patients might need to be ameliorated with intravenous immunoglobulin and additionally adding immunosuppressive agents such as cyclosporine 62.

The following steps are recommended in the processing of hem-irAEs: 1. Refine the hematology-related laboratory tests, including Prothrombin time, Activated partial thromboplastin time, and fibrin level, which are helpful to rule out the occurrence of a potential DIC (Disseminated Intravascular Coagulation). Further hematological tests including blood smear, reticulocyte count, hemolysis-related biochemistry (lactate dehydrogenase, bilirubin, and haptoglobin), and the direct antiglobulin test (DAT) should be performed to clarify the mechanism of anemia. In cases of cytopenia with hyperthermia (above 39-40 degrees Celsius), the development of cytokine release syndrome with hemophagocytic syndrome needs to be monitored and further laboratory tests, including ferritin, triglycerides and fibrinogen, are required for identification. 2. Bone marrow analysis is occasionally required to distinguish whether cytopenia is of central or peripheral origin. 3. Exclude cytopenia caused by the participation of other drugs under the guidance of drug regulatory authorities. 4. Using serological tests or PCR tests to exclude viral infections, such as HIV, hepatitis C, HSV1/2, CMV, varicella zoster virus, EBV, etc. 5. Be alert to primary hematologic tumors and bone metastases from solid tumors, blood smear examination, bone marrow infiltration and immunophenotyping of circulating lymphocytes should be performed. 6. Search for potential autoimmune diseases, such as systemic rheumatoid arthritis, lupus erythematosus, autoimmune endocrinopathies, cutaneous autoimmunity, etc. Anti-nuclear antibody tests or anti-DNA antibody tests can be used to identify autoimmune diseases 63.

Once hem-irAE is diagnosed, appropriate treatment needs to be administered timely. Some asymptomatic episodic leukocyte count reductions do not require specific treatment, while under certain circumstances of episodic bleeding, fatigue, neutropenia close to 0/mm3, high fever, and certain infections, patients are encouraged to be hospitalized for careful observation and vigorous prevention of infections. The bleeding can be treated with a Full dose of corticosteroids (1 mg/kg/day) for three consecutive weeks. Adding intravenous immunoglobulin to steroids when the bleeding index rises above 8 64. Rituximab or thrombopoietin agonists can be used synergistically to increase the body's response to steroids. For the management of anemia, GCSF (Granulocyte Colony-Stimulating Factor) can enhance the production of neutrophils effectively. Cyclosporine or thrombopoietin agonists are considered in cases of inadequate response to corticosteroids, and anti-lymphocyte serum should be considered for patients without comorbidities. For neutropenia, CSF combined with antibiotics is recommended until the white blood cell levels are up to normal, whereas corticosteroids should not be given systematically since they raise the risk of infection 65. For hemolytic anemia, the recommended dose of corticosteroids is 1.5 mg/kg/day for 15 days and then tapered, with red blood cell transfusion support if necessary 66. For cytopenia with hyperthermia, a high dose of corticosteroids (3-5 mg/kg/day) plus anti-IL-6 (tocilizumab or siltuximab) is required. A single dose of etoposide 150 mg/kg IV is considered in case of inadequate response 67. In general, during the management of hem-irAEs, except for asymptomatic eosinophil count increase, during which ICIs can be continued under close observation, ICI therapy should be considered suspended regardless of the grade of irAE, or the resumption of immunotherapy should be carefully chosen after the condition is under well control.

### 2.2 Dermatologic toxic effects

Rash and pruritus are the most common dermatologic toxic effects of ICIs in combination therapy for breast cancer, with approximately 40% of the patients experiencing this irAE. Fortunately, the rate of grade 3-4 skin reactions during treatment with ICIs remains low, ranging from 1%-3.8%, occurring mainly with PDL1 inhibitors, which is a decrease from 10% in the previous trials 68, 69 (**Figure 4B**). This may account for the earlier use of corticosteroid drugs for control by current clinicians. The clinical manifestation of mild skin toxicity is a rash with no obvious clinical symptoms or a rash with pruritus. The rash is mainly reticular, erythematous, edematous, and maculopapular, often occurring on the trunk and extremities 70, 71. Grade 1-2 rash or pruritus is relatively more manageable by topical corticosteroids, cold compresses, and oatmeal baths. As the symptoms progress to a severe state, oral corticosteroids are required. In addition to this, additional immunosuppressive medications, such as infliximab, mycophenolate mofetil, or cyclophosphamide, are also considered. The ICIs treatment should be suspended until AEs are downgraded to grade1-2. If the skin toxicity does not diminish after up to 12 weeks of supportive therapy, permanent termination of immunotherapy must be taken into consideration 72. Other than Rash and pruritus, dermatologic toxic effects that may occur with the use of ICIs are bullous pemphigoid, Sweet syndrome, Stevens-Johnson syndrome/toxic epidermal necrolysis, Mucosal toxic effects, and Vitiligo, which are fairly rare and more often found in anti-PD-L1 antibodies 73. Most of the symptoms can be managed with supportive care including topical steroids, viscous lidocaine hydrochloride, and good hygiene, except for the development of Stevens-Johnson syndrome/toxic epidermal necrolysis or Vitiligo, for which clinicians should consider permanently discontinuing treatment with ICIs 74.

### 2.3 Pneumonitis

Checkpoint inhibitor pneumonia (CIP) is one of the most hazardous adverse effects in oncology patients receiving ICI therapy. The clinical manifestations are not specified including dyspnea, cough, fever, chest pain, and decreased exercise tolerance. Pneumonia is an irAE that can have life-threatening effects despite its low incidence 75. According to the World Health Organization Drug Alert database, of the 613 fatalities resulting from ICI treatment from 2018 to 2019, 35% of which were due to CIP caused by anti-PD-1/PD-L1 drugs 76. Studies have shown that the incidence of CIP correlates with different types of ICI. A pan-cancer study by Su showed that all kinds of anti-PD-1/PD-L1 drugs increase the incidence of grade 1 to 5 pneumonia. Among them, pembrolizumab is inclined to increase grade 3-5 pneumonia 77. In breast cancer, the incidence of pneumonia in combination therapy for breast cancer ranges from 1-7%, which is higher in patients receiving anti-PD-1 therapy compared with anti-PD-L1 therapy alone or in combination (**Figure 3C**) (**Figure 4C**). Although the proportion of patients with severe pneumonia is less than 3%, there are many reported cases of pneumonia leading to death in the treatment of NSCLC 78. There were no deaths due to pneumonia have been reported in the treatment of breast cancer, while two cases of discontinuation of ICIs due to severe pneumonia have been reported 79. As a result, pneumonia-related surveillance is extremely important. Any patient presenting with symptoms associated with pneumonia such as upper respiratory tract infection, cough, shortness of breath, or hypoxemia should be alerted to the development of pneumonia and require further imaging to clarify the diagnosis, such as a CT scan. CIP has Multiple imaging presentations. Statistically, Naidoo found that there are 19% organic pneumonia (OP), 37% ground-glass opacity (GGO), 22% acute interstitial pneumonia (AIP), 7% hypersensitivity pneumonitis (HP), and 5% unclassified pneumonia 80. Due to the limited fiberoptic bronchoscopy on specimen acquisition, few relevant reports described the pathological types of CIP. Of 9 patients with CIP reported by Larsen, 7 with organic pneumonia, 3 with combined occult febrile necrotic airspace granuloma, 1 with acute fibrinous pneumonia, and 1 with diffuse alveolar injury, all 9 with foamy macrophages and pulmonary cell vacuolization 81. Recommendations for the diagnosis and treatment of CIP are relatively limited as there are no prospective trials to evaluate the optimal treatment strategy for CIP. In moderate to severe cases, a bronchoscopy should be performed to rule out an infectious etiology before starting immunosuppressive therapy. Corticosteroids are the mainstay of treatment for CIP. Current guidelines recommended 1 mg/(kg. d) prednisone for grade 2 CIP and 2-4 mg/ (kg. d) prednisone for grade 3-4 CIP. Patients are considered to have steroid-refractory CIP when there is no clinical improvement after 48-72 h of hormone therapy. Patients should be hospitalized and treated with or without additional immunosuppression, including mycophenolate, cyclophosphamide, and infliximab 82. No further immune checkpoint inhibitors should be administered.

### 2.4 Hepatitis

In combination with ICIs therapy, hepatitis is mainly manifested by abnormal liver function, including alanine aminotransferase increased, aspartate aminotransferase increased, and occasionally bilirubin increase (**Figure 3C**) (**Table 6**). A case of death due to autoimmune hepatitis was reported in Impassion130^22^. In addition to this, the incidence of grade 3-4 hepatitis in combination therapy ranges from 0.5%-5.2% (**Figure 4D**). Since most liver function abnormalities are asymptomatic, clinicians are urged to closely monitor patients' liver function during ICIs therapy. Most of the patients obtained hepatitis 8-12 weeks since the immunotherapy began. More patients who received anti-PD-1 antibodies were prone to abnormal liver function compared to anti-PD-L1 antibodies and more often in PD-1 combined with CTLA4 therapy 18, 69. When patients develop mild abnormalities in liver function during immunotherapy, it is necessary to exclude the possibility of other drugs or viral-related hepatitis 14. If other causes are eliminated, prompt administration of corticosteroids (prednisone 1-2-mg/kg/d or methylprednisolone 0.5-1-mg/kg/d) is recommended. Liver function should be tested every 3-5 days until AE is back to grade 1 or the measures return to normal. If the patient develops grade 3-4 immune hepatitis, the patient should be terminated ICIs permanently and receive intravenous methylprednisolone 1-2 mg/kg daily with liver function tests every 1-2 days and hormone reduction maintained for more than 1 month. When severe conditions arise, such as when steroids fail to elevate ALT/AST, mycophenolate (500-1000 mg every 12 hours) or tacrolimus (500-1000 mg every 12 hours) may be helped 83, 84.

### 2.5 Diarrhea and Colitis

Diarrhea and colitis are more common and less symptomatic in the treatment of immune agents in combination with chemotherapy (**Figure 3C**). Along with the rising rate of immunosuppressant use, the incidence of immune-related gastrointestinal injury is also increasing over the years. The clinical manifestations include abdominal pain, diarrhea, bloody stools, weight loss, fever, etc. It can also be accompanied by a variety of extraintestinal manifestations, such as arthralgia, endocrine abnormalities, skin damage, hepatitis, nephritis, and pericarditis 85. According to the National Cancer Institute of America, the severity of diarrhea and colitis is graded as follows: Grade 1 diarrhea is defined as having less than 4 bowel movements per day above baseline; Grade 2 diarrhea is defined as having 4-6 bowel movements per day above baseline and Grade 2 colitis is defined as abdominal pain or mucus and blood stools; Grade 3 diarrhea is defined as having 7 or more bowel movements per day above baseline and Grade 3 colitis is defined as having severe abdominal pain, signs of peritoneal irritation, fever and other symptoms of intestinal obstruction or intestinal perforation; Grade 4 is defined as having severe or even life-threatening symptoms^86^. Laboratory tests may show elevated C-reactive protein, anemia, hypoalbuminemia, and some patients may have positive autoimmune antibodies (e.g., Anti-neutrophil plasma antibodies) 87.

Diarrhea caused by checkpoint inhibitor therapy is thought to be a consequence of underlying colonic inflammation colitis (IMC). The incidence of grade 3/4 diarrhea and colitis are not significantly higher among patients treated with the PD-1/PD-L1 inhibitors compared with chemotherapy monotherapy (**Figure 4E**). It is necessary to rule out any viral or bacterial infections when a patient develops diarrhea during immunotherapy since immunosuppression rise the chance for subsequent opportunistic infections 88, 89. Cytomegalovirus and Salmonella infections have been reported to coexist with IMC 90. In addition to infectious factors, the presence of metastatic lesions in the gastrointestinal tract needs to be identified. Colonoscopy and mucosal biopsy are helped to diagnose IMC precisely in patients with grade 2 or higher persistent diarrhea. Endoscopically IMC usually shows ulcers, erosions, erythema, loss of vascular texture, and bleeding 91. Intestinal mucosal biopsies frequently show acute inflammation and, in rare cases, chronic inflammation, with very exceptional cases of lymphocytic colitis (i.e., >20 lymphocytes/10 epithelial cells) 92.

Antidiarrheal agents can be used to intervene in patients with mild diseases when other non-immunosuppression-related etiologies have been excluded. Immunotherapy can be suspended until the symptoms have subsided. Generally, there is no need to terminate immunotherapy because of diarrhea or colitis. A retrospective study from MD Anderson showed an increased need for infliximab on top of corticosteroid therapy for grade 2 and higher diarrhea (97% vs. 73%), but no difference in the need for immunosuppressive therapy on different grades of colitis. It is worth mentioning that the study also found that patients presenting with ICI- induced diarrhea or colitis had better OS rates, which indicated that diarrhea is an independent predictor of improved survival, independent of treatment 86.

### 2.6 Endocrinopathy

Immune-related endocrine disorders are usually delayed and persistent, influenced by the type, dose, and combination therapy of ICI 93. Common immune-related endocrine disorders include abnormal thyroid function, autoimmune diabetes mellitus, pituitary inflammation, and primary adrenocortical insufficiency. Some of these appear after 6-7 weeks of dosing while the majority occur within 12 weeks of treatment initiation. This type of irAE is more prevalent but also is difficult to diagnose because of the complex clinical signs and poorly characterized features. Patients may present with numerous non-specific symptoms, including fatigue, nausea, headache, and depression 94. Thyroid function abnormalities are the most frequently reported endocrine gland irAEs, which can manifest as hypothyroidism, hyperthyroidism, and acute thyroiditis. Studies have shown that PD-L1 inhibitors cause a higher incidence of hyperthyroidism and hypothyroidism than PD-1 inhibitors, and combination therapy can lead to an increased incidence of thyroid dysfunction 95, 96. Based on case reports and expert consensus, regular monitoring of thyroid function in patients receiving immunotherapy is recommended, including the detection of morphological abnormalities in the thyroid gland by thyroid ultrasound, and thyroid-related autoantibody testing. According to the European Society for Endocrinology Guideline, radioactive iodine uptake rate measurements are helpful for differential diagnosis 97. The use of beta-blockers is recommended for thyrotoxicosis and the use of thyroid hormone replacement therapy for hypothyroidism 98. Most patients with abnormal thyroid function can be corrected in the short term with appropriate treatment and the ICIs generally do not need to discontinue.

When the ICIs drugs affect the endocrine function of the pancreas, it leads to the development of autoimmune diabetes mellitus. The clinical features of autoimmune diabetes mellitus are similar to those of fulminant type I diabetes mellitus, including rapidly increasing blood glucose, often leading to ketoacidosis, lack of endogenous insulin, and low or undetectable C-peptide levels 99. It is recommended to monitor blood glucose. For patients with ketoacidosis, it is suggested to correct water loss, restore blood volume by rehydration, and adjust blood glucose by continuously pumping low-dose insulin intravenously. The destruction of pancreatic B cells in patients with autoimmune diabetes is tissue-specific and the autoimmune response is terminated when the destruction is terminated, so there is no need to use hormones to suppress the autoimmune response 93.

The development of pituitary inflammation because of PD-1/PD-L1 inhibitors is common and has been reported in clinical trials for a variety of cancers. Also, combination therapy has been shown to be associated with higher rates of pituitary inflammation, with rates ranging from 7.7% to 10.5% 100. The main manifestation is hypopituitarism, which manifests as a deficiency of ACTH, thyrotropin, and gonadotropin, and clinically manifests as loss of appetite, weakness, nausea, hyponatremia, hypotension, and hypoglycemia. MRI of the pituitary gland in the acute phase may show enlargement of the pituitary gland and may also exclude pituitary tumors or metastases 101. It is suggested to conduct glucocorticoid replacement therapy under the close monitoring of hormone levels. For patients receiving ICIs who present with pituitary inflammation, PD-1/PD-L1 inhibitors are not an absolute contraindication to use, and ICIs-like drugs may be suspended during the acute phase. Treatment may be restarted when appropriate glucocorticoid replacement therapy has been administered until the condition has stabilized 15.

Endocrine diseases usually require permanent hormone replacement therapy after onset due to their irreversible nature 102. The most common endocrine disorders during immune combination therapy remain pituitary inflammation and hypothyroidism, which is consistent with previous studies 102. Apart from those, diabetes and hyperthyroidism have also been reported in some literature 30, 31. Based on reports of other types of cancer, the rate of immunotherapy causing endocrine disease is less than 1% in the treatment of advanced melanoma 42, 103 and 12% in the treatment of non-small cell lung cancer 104. Yet current clinical data show a 10-20% incidence of endocrine disease associated with immunotherapy in the treatment of breast cancer (**Figure 3D**) (**Figure 4F**). It appears that patients with breast cancer are more likely to develop endocrine system disorders while receiving immunotherapy. Moreover, a case of hyperthyroidism developing into pituitary inflammation was reported in the combined treatment of breast cancer 32. The diagnosis of pituitary dysfunction is somewhat dicer and relies on some biochemical evidence such as low adrenocorticotropic and thyrotropin and occasionally low luteinizing hormone and/or prolactin, usually requiring peripheral blood evaluation 94.

## Conclusion and perspective

The use of immunotherapy in breast cancer continues to gain momentum with the release of results from various clinical trials, and its efficacy has been recognized. Effective management of immune-related adverse reactions is an equally important part of the immunotherapy process. In this review, we found that in breast cancer immunotherapy, adverse immune reactions are characterized by a high incidence, mostly self-limiting, and mostly minor. Although breast cancer patients are often treated with a combination of therapies, when immunotherapy is combined with other therapies, the safety profile is relatively manageable. When a mild irAE occurs, currently the clinician's strategy remains symptomatic treatment and hormone therapy. Whereas clinicians often have no choice but to terminate immunotherapy when some of the moderate-to-severe immune adverse events occur. As a result of the termination of immunotherapy, the treatment is stalled and the tumor disease may progress, along with the patient's body being devastated by severe adverse effects. Therefore, this research team believes that the current Landscape for adverse immune reactions is largely clear and that the next step in the research should focus more on how to prevent adverse immune reactions from occurring. On the one hand, researchers can try to narrow down the population for immunotherapy, so that the optimal beneficiaries of immunotherapy can be screened prior to immunotherapy, and those who are likely to be hit by severe adverse immune reactions can be excluded in advance. On the other hand, researchers can try to develop immunotherapeutic drugs that work more precisely on the primary lesion and reduce the damage of immunosuppressive drugs on other organs. More relevant experiments and clinical trials are warranted.

## Figures and Tables

**Table 1:** The Anti-PD1/PDL1 Antibodies used as ICIs for Oncology Treatment

Drug	Medication number	Commercial name	Target	Indication
Pembrolizumab	MK3475	Keytruda	PD-1	SKCM, NSCLC, HL, HNSCC, BLCA, STAD
Nivolumab	ONO4538	Opdivo	PD-1	SKCM, NSCLC, RCC, BLCA, COAD, HCC, HNSCC
Cemiplimab	REGN2810	Libtayo	PD-1	CSCC, BCC, NSCLC
Toripalimab	JS001	-	PD-1	SKCM, HNSCC
Sintilimab	IBI308	-	PD-1	HL, NSCLC, HCC, COAD
Tislelizumab	BGB-A317	-	PD-1	HL
Camrelizumab	SHR-1210	-	PD-1	HL, HCC, NSCLC, ESCA
Atezolizumab	MPDL3280A	Tecentriq	PD-L1	BLCA, NSCLC, SCLC, TNBC, HCC, SKCM
Durvalumab	MEDI4736	Imfinzi	PD-L1	NSCLC, ES-SCLC, BLCA
Avelumab	MSB0010718C	Bavencio	PD-L1	MCC, BLCA, RCC

**Table 2:** The study characteristic of the combination therapy in unresectable, locally advanced, or metastatic breast cancer

Study	Year; Author	Phase	Sample size	Target molecule	Chemotherapeutic agents involved	Follow-up(months)	Efficacy
Impassion 130	2021; Emens	III	902	Atezolizumab	nP	18.8	PFS and OS benefit in PD-L1 IC+ population
Impassion 130 (JP)	2019; Iwata	III	65	Atezolizumab	nP	18.8	PFS and OS benefit in PD-L1 IC+ population
Impassion 131	2021; Miles	III	651	Atezolizumab	paclitaxel	9	Negative outcome of PFS in PD-L1+ population or the whole populaiton
KEYNOTE 355	2020; Cortes	III	847	Pembrolizumab	nP paclitaxel gemcitabine plus carboplatin	25.9	PFS benefit in CPS≥10 population
GP28328	2018; Adams	1b	33	Atezolizumab	nP	24.4	manageable safety profile
-	2020; Tolaney	II	90	Pembrolizumab	eribulin	10.5	Negative outcome of PFS in PD-L1+ or the ITT populations

JP = the subgroup analysis of Japan; nP = nab-paclitaxel; PFS = progression-free survival; OS = overall survival; IC+ = immune cell-positive (tumours with ≥1% PD-L1 expression); CPS = combined positive score; ITT = intention-to-treat

**Table 3:** The Study Characteristics of the Combination Therapy in NACT

Study	Year; Author	Study design	Phase	Sample size	Target molecule	Median follow-up	pCR
Gepar Nuevo	2018; Loibl	multicenter, prospective, randomized, double-blind, placebo-controlled	II	174	durvalumab	NA	dur vs P53.4% vs44.2%
Impassion 031	2020; Mittendorf	randomized, multicenter, multinational, double-blind	III	333	atezolizumab	20.6	A vs P58% vs 41%
KEYNOTE522	2020; Schmid	randomized, double-blind	III	602	pembrolizumab	15.5	Pem vs P64.8 %vs 51.2%
I-SPY2	2020; Nanda	multicenter, open-label, adaptively	II	69	pembrolizumab	33.6	Pem vs control 44% vs17%(HER2-) 30% vs13%(HR+/HER2-)60% vs 22%(TNBC)

TNBC = triple-negative breast cancer; pCR = pathologic complete response; dur = durvalumab; P = placebo; A = atezolizumab; Pem = pembrolizumab; HR+ = hormone receptor-positive; HER2- = human epidermal growth factor receptor 2-negative; NA = not available

**Table 4:** Ongoing Clinical Trails Focus on Evaluating a Combination of PD1/PD-L1 Inhibitors and CTLA4 Inhibitors in Breast Cancer (May 2022)

NCT	Study	Phase	Condition/disease	Intervention/treatment
NCT03132467		I	hormone receptor-positive, HER2 negative stage II-III breast cancer	durvalumab and tremelimumab
NCT03409198	ICON	II	Hormone Receptor-Positive TumorMetastatic Breast Cancer	Drug: IpilimumabDrug: NivolumabDrug: Pegylated liposomal doxorubicinDrug: Cyclophosphamide
NCT03982173	MATILDA	II	Triple Negative Breast Cancer	Drug: TremelimumabDrug: Durvalumab
NCT03789110	NIMBUS	II	hypermutated HER2 negative breast cancer	Drug: NivolumabDrug: Ipilimumab
NCT03518606	MOVIE	I/II	advanced Breast Cancer	Drug: Durvalumab + Tremelimumab + metronomic Vinorelbine
NCT04185311		I	triple-negative or estrogen receptor-positive, HER2 negative localized breast cancer	Biological: IpilimumabBiological: NivolumabBiological: Talimogene Laherparepvec
NCT02643303		I/II	advanced Breast Cancer	Drug: DurvalumabDrug: TremelimumabDrug: Poly ICLC

PFS = progression-free survival

**Table 5:** The clinical presentation of irAEs by organ type

AEs	Clinical presentation
Hematologic toxicity	Neutropenia, febrile neutropenia, anemia
Dermatologic toxicity	Rash, pruritus, vitligo
Pneumonitis	Cough, dyspnoea, fever, chest pain
Hepatitis	Asymptomatic, fatigue, nausea/vomiting, ALST/ASATincreased
Colitis	Diarrhea, abdominal pain, pseudo-obstruction, fever, weight loss
Endocrinopathy	Hypothyroidism, hyperthyroidism, diabetes, pituitary inflammation, adrenal insufficiency

**Table 6:** Grading of immune-related hepatotoxicity

Specific description
AsymptomaticALT or AST ≤2.5×ULNTotal bilirubin≤1.5×ULN
2.5×ULN<ALT or AST≤5×ULN1.5×ULN<total bilirubin≤3×ULN
ALT or AST >5×ULNTotal bilirubin>3×ULN

## Abbreviations

PD-1: Programmed cell death 1
PD-L1: Programmed cell death ligand 1
irAEs: immune-related adverse effects
CTLA4: cytotoxic T lymphocyte-associated protein 4
IARC: International Agency for Research on Cancer
ICIs: immune checkpoint inhibitors
APC: antigen-presenting cell
TME: tumor microenvironment
QOL: quality-of-life rating
FDA: Food and Drug Administration
PFS: progression free survival
ITT: intent-to-treat
OS: overall survival
CPS: comprehensive positive score
ORR: overall response rate
AR: androgen receptor
CBR: clinical benefit rate
PARP: poly adenosine diphosphate-ribose polymerase
CR: complete response
SD: stable disease
PD: progressive disease
CTCAE: Common Terminology Criteria for Adverse Events
DIC: Disseminated Intravascular Coagulation
DAT: direct antiglobulin test
GCSF: Granulocyte Colony-Stimulating Factor
CIP: Checkpoint inhibitor pneumonia
OP: organic pneumonia
GGO: ground-glass opacity
AIP: acute interstitial pneumonia
HP: hypersensitivity pneumonitis
IMC: inflammation colitis
SKCM: Skin cutaneous melanoma
NSCLC: Non-small-cell lung cancer
HL: Hodgkin's lymphoma
HNSCC: Squamous-cell carcinoma of the head and neck
RCC: Renal cell carcinoma
HCC: Hepatocellular carcinoma
CSCC: Cutaneous squamous cell carcinoma
SCLC: Small cell lung cancer
TNBC: Triple-negative breast cancer
ES-SCLC: Extensive stage-small cell lung cancer
MCC: Merkel cell carcinoma
ESCA: Esophageal Cancer
BLCA: Bladder Urothelial Carcinoma
STAD: Stomach adenocarcinoma
COAD: Colon adenocarcinoma
BCC: Basal cell carcinoma
